# Interface microstructure effects on dynamic failure behavior of layered Cu/Ta microstructures

**DOI:** 10.1038/s41598-023-37831-5

**Published:** 2023-07-13

**Authors:** Rajesh Kumar, Jie Chen, Avanish Mishra, Avinash M. Dongare

**Affiliations:** 1 Department of Materials Science and Engineering, Institute of Materials Science, University of Connecticut, 97 North Eagleville Road, Unit 3136, Storrs, CT 06269-3136 USA; 2 Present Address: Department of Mechanical Engineering, National Institute of Technology Hamirpur, Hamirpur, India; 3 Present Address: Theoretical Division (T-1), Los Alamos National Laboratory, Los Alamos, NM USA

**Keywords:** Atomistic models, Mechanical properties

## Abstract

Structural metallic materials with interfaces of immiscible materials provide opportunities to design and tailor the microstructures for desired mechanical behavior. Metallic microstructures with plasticity contributors of the FCC and BCC phases show significant promise for damage-tolerant applications due to their enhanced strengths and thermal stability. A fundamental understanding of the dynamic failure behavior is needed to design and tailor these microstructures with desired mechanical responses under extreme environments. This study uses molecular dynamics (MD) simulations to characterize plasticity contributors for various interface microstructures and the damage evolution behavior of FCC/BCC laminate microstructures. This study uses six model Cu/Ta interface systems with different orientation relationships that are as- created, and pre-deformed to understand the modifications in the plasticity contributions and the void nucleation/evolution behavior. The results suggest that pre-existing misfit dislocations and loading orientations (perpendicular to and parallel to the interface) affect the activation of primary and secondary slip systems. The dynamic strengths are observed to correlate with the energy of the interfaces, with the strengths being highest for low-energy interfaces and lowest for high-energy interfaces. However, the presence of pre-deformation of these interface microstructures affects not only the dynamic strength of the microstructures but also the correlation with interface energy.

## Introduction

Multiphase metallic materials (M^3^s) show promise for applications under extreme environments due to improved strength, hardness, toughness, and resistance to corrosion and radiation^1–7^. Of particular importance are FCC/BCC layered microstructures with nanoscale distributions of interfaces that provide an enhanced response compared to the coarse-grained counterparts. For example, recent experimental capabilities^8,9^ have enabled the fabrication of several FCC/BCC multilayered microstructures with control over the interface spacing and structure. The orientation relationships (OR) and spacings determine the plasticity contributions that affect the response under mechanical, thermal, and radiation extremes^4–7,10–15^. These interfaces can serve as a barrier, source, or sink for dislocations and affect the dynamic properties of the multiphase microstructures. In addition, the modifications in interface structure due to fabrication methods (flat^16^ vs. faceted^17^) result in modifications in the deformation modes of the FCC phase. In addition, incoherent interfaces can render improved tensile strengths as they can act as barriers to dislocation slip and lead to dislocation pile-ups at the interface^15^. Thus, the interface microstructure (structure and OR) affects the plasticity contributions and their evolution behavior and hence the deformation behavior of layered multiphase microstructures^18–23^.

However, the effect of FCC/BCC interface microstructure on the failure behavior due to nucleation/evolution of voids (spallation) is still not clearly understood. For example, while voids are likely to nucleate at interfaces, the spall failure of Cu/Nb alloys suggests that voids nucleate in the Cu phase and not at the interface^24^. The challenge in understanding the role of interface microstructure is the inability to experimentally characterize the evolution of defects that contribute to void nucleation behavior. Molecular dynamics (MD) simulations can characterize these atomic scale mechanisms for various loading conditions^25–32^. For FCC/BCC interfaces, MD simulations have investigated the role of the structure, OR, and spacing on the mechanisms of plastic deformation and strengthening behavior of laminate microstructures^10,12,15,18,20,22,23,33–46^. The investigation of dynamic failure behavior of FCC/BCC interfaces using MD simulations is limited mainly to the role of interface spacing ^47,48^ which suggests void nucleation at the interface for spacings of 6 nm and lower and in the bulk of the FCC layers and at the interface for spacings of 12 nm and larger. Most of these studies, however, are carried out for loading perpendicular to the interface. The defect evolution and void nucleation behavior for loading parallel to the interface in these materials is still unclear.

This manuscript aims to investigate the role of interface microstructure in layered Cu/Ta systems on the void nucleation stresses/strains at the onset of dynamic failure using MD simulations. The loading conditions experienced by systems undergoing spall failure comprise shock compression and shock release, followed by triaxial tension (uniaxial strain), wherein voids nucleate and grow/coalesce to form cracks^49^. Application of uniaxial strain/expansion in one direction results in a triaxial state of stress. MD simulations mimic these loading conditions using uniaxial strain expansion during spallation using constant strain rate loading conditions of 10^9^ s^−1^. The angular-dependent potential (ADP)^50^ is used for the interatomic interactions in the Cu/Ta system. The as-created systems are deformed in the direction perpendicular to the interface to understand the plasticity contributors (from dislocations like Shockley partial, Frank, stair-rods and twinning partial etc. and twins) at the onset of spall failure. The Cu/Ta layered systems to study interface microstructures use the “Kurdjumov–Sachs” (KS)^51^, “Nishiyama-Wassermann” (NW)^52^, KS112^53^, and some other ORs^11^. These interface microstructures render modifications in the density of dislocations and hence the energy that affects the plasticity contributors and damage evolution behavior during spall failure. The microstructure of the interface for the various ORs considered here and their computed energies are tabulated in Table S1 of the Supplemental Information. The Cu/Ta systems are also pre-deformed under uniaxial strain compression and unloaded to zero pressure to distribute dislocations in the microstructure (referred to as the ‘pre-deformed’ systems). The pre-deformed systems are then subjected to uniaxial strain tension to understand the modifications in plasticity contributors and the mechanisms and stresses for void nucleation. In addition, the simulations investigate the plasticity contributors, and the role of the void nucleation stresses for loading in the direction parallel to the interfaces.

## Results and discussion

### Plasticity contributors for void nucleation in Cu/Ta laminates with KS interface

**Figure 1:**
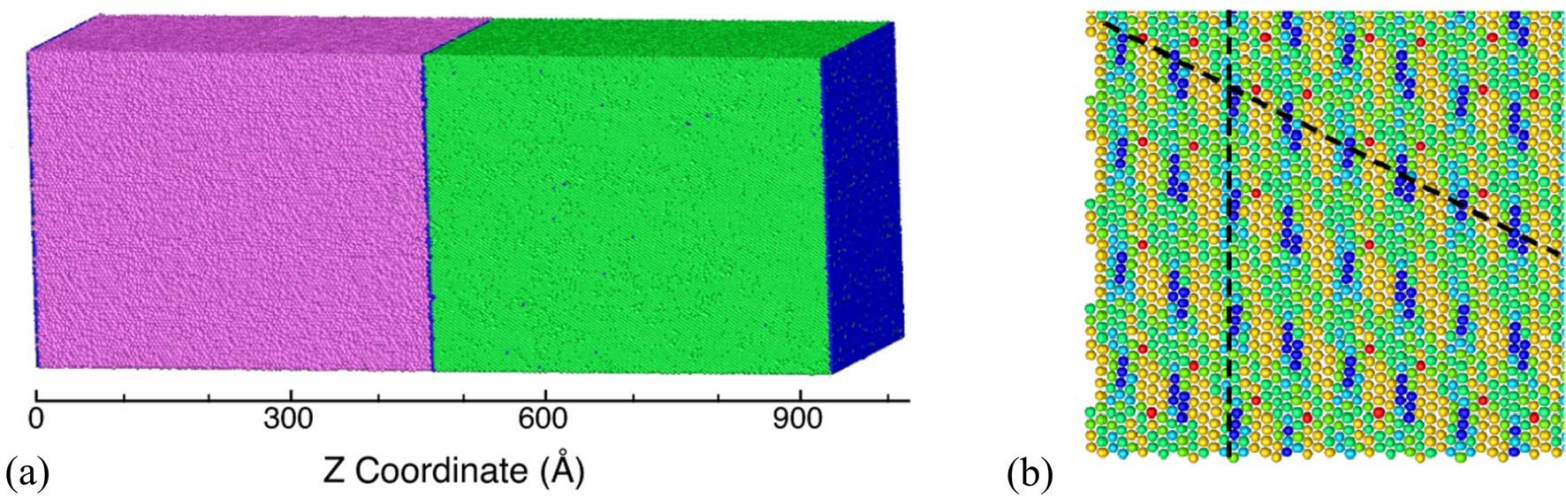
(**a**) Initial microstructure of the layered Cu/Ta system with the KS orientation relationship. Here, purple, green, blue, red, and orange color atoms represent Ta-BCC, Cu-FCC, disordered, stacking fault, and Cu/Ta surface/void atoms, respectively. (**b**) The corresponding structure of the interface with the Z-stress values coloring the atoms. Here the misfit dislocations are shown by the dashed line.

**Figure 2:**
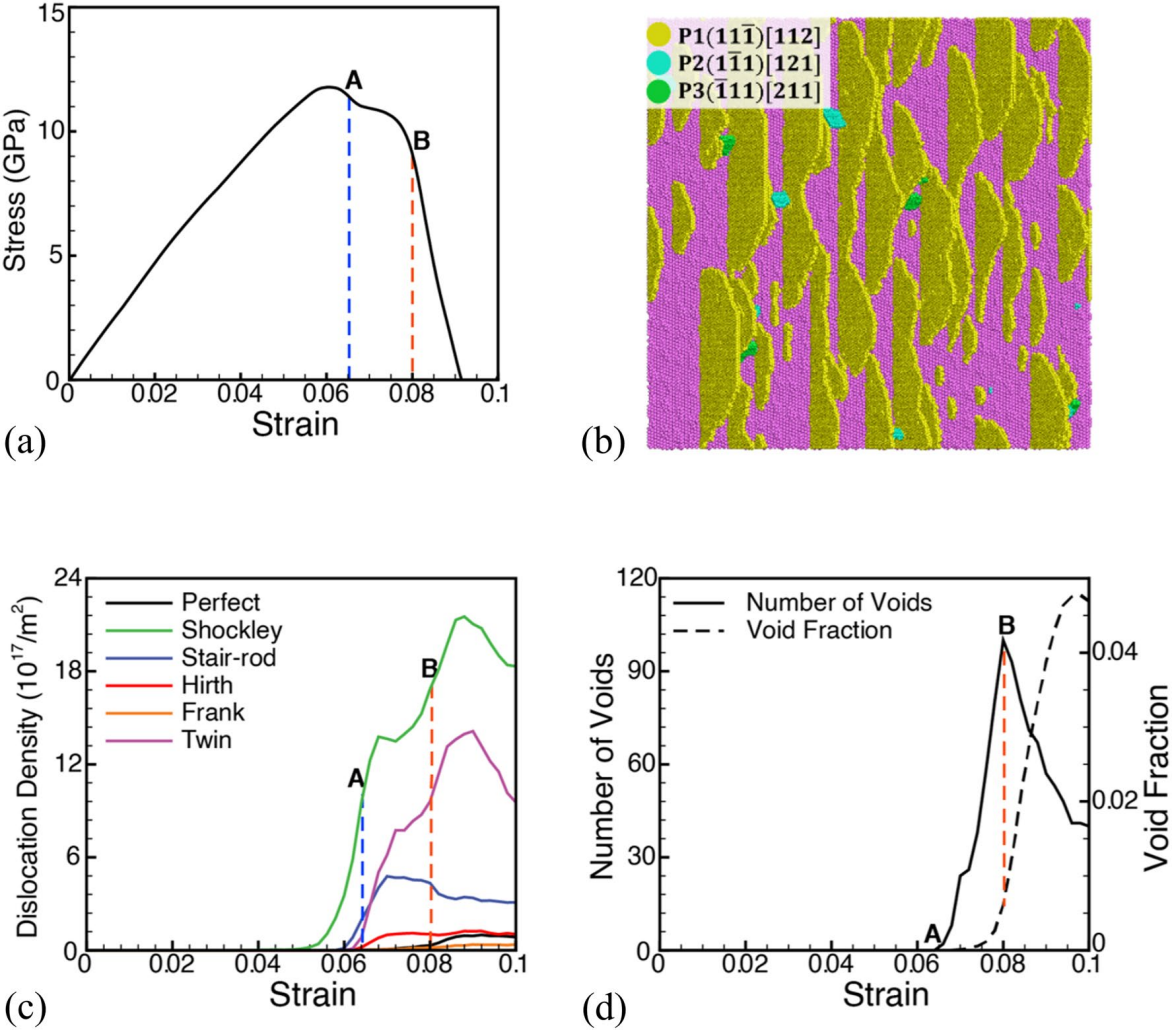
(**a**) Stress–strain plot for deformation of the Cu/Ta system in the direction perpendicular to the KS interface. (**b**) Distributions of stacking faults at the KS interface showing the plasticity contributions from different slip systems. Plots (**c,d**) show the evolution of dislocation density and the number of voids, respectively. Here, blue and orange dashed vertical lines at points A and B represent the void nucleation and peak number of voids points, respectively.

The study first investigates the plasticity contributors during the deformation of the layered KS interface Cu/Ta microstructures with the lowest interface energy under uniaxial tensile strain loading perpendicular to the interface. Figure 1a shows the initial Cu/Ta KS interface microstructure wherein purple, green, and blue atoms represent the BCC Ta phase, FCC Cu phase, and disordered (interface) atoms, respectively. The KS interface results in misfit dislocations distributed along the interface marked, as shown in Fig. 1b by the dashed black lines. The stress–strain response of the KS interface of Cu/Ta under uniaxial expansion is plotted in Fig. 2a. The plot shows a linear increase in stress (elastic region) followed by a small deviation due to plasticity (dislocations). Plasticity in layered microstructures is observed through heterogeneous nucleation of dislocations from the interface, as shown in Fig. 2b. The continued deformation of the system shows two drops in stress values wherein the first drop (labeled as point A) in stresses is gradual and is followed by a second and relatively sharp drop (labeled as point B) in stresses during deformation. The corresponding evolution of the density of Cu dislocations is plotted in Fig. 2c. Plasticity for the system is only observed in the Cu phase; hence, the dislocations characterized are only for the Cu layer. The plot shows plasticity initiates well before point A. Shockley partials dominate slip, followed by twinning partials and perfect dislocations. The snapshots are characterized to quantify the evolution of the number of voids and the void fraction under these loading conditions and the evolution of the number of voids and void volume fraction as plotted in Fig. 2d. The dashed-blue line at point A represents the stress/strain corresponding to the nucleation of voids, and the dashed-red line at point B represents the peak number of voids nucleated, after which additional strain results in the growth/coalescence of existing voids. The stair-rod partials are observed to be at a peak during this void nucleation stage and can be expected to contribute by providing void nucleation sites in the Cu phase^32,54,55^.

Thus, a “nucleation stage” of voids is identified between points A (nucleation of 1^st^ void) and B (peak number of voids), followed by a “growth and coalescence” stage that results in a steady decrease in the number of voids in the system along with an increase in void fraction to form a crack and initiate failure. Failure is defined as when the applied stress reaches zero. The plots are used to quantify a ‘maximum void nucleation stress’, ${\sigma }_{V}^{M}$, (11.4 GPa) and a ‘minimum void nucleation stress’, ${\sigma }_{V}^{N}$, (9.1 GPa) wherein voids are observed to nucleate. Similarly, a ‘maximum void nucleation strain’, ${\varepsilon }_{V}^{M}$, (0.08), and a ‘minimum void nucleation strain’, ${\varepsilon }_{V}^{N}$**,** (0.066) can be quantified for this interface and loading conditions. Thus, the drop in stresses at point A and B are attributed to the nucleation and evolution of voids.

#### Effect of pre-deformation

**Figure 3:**
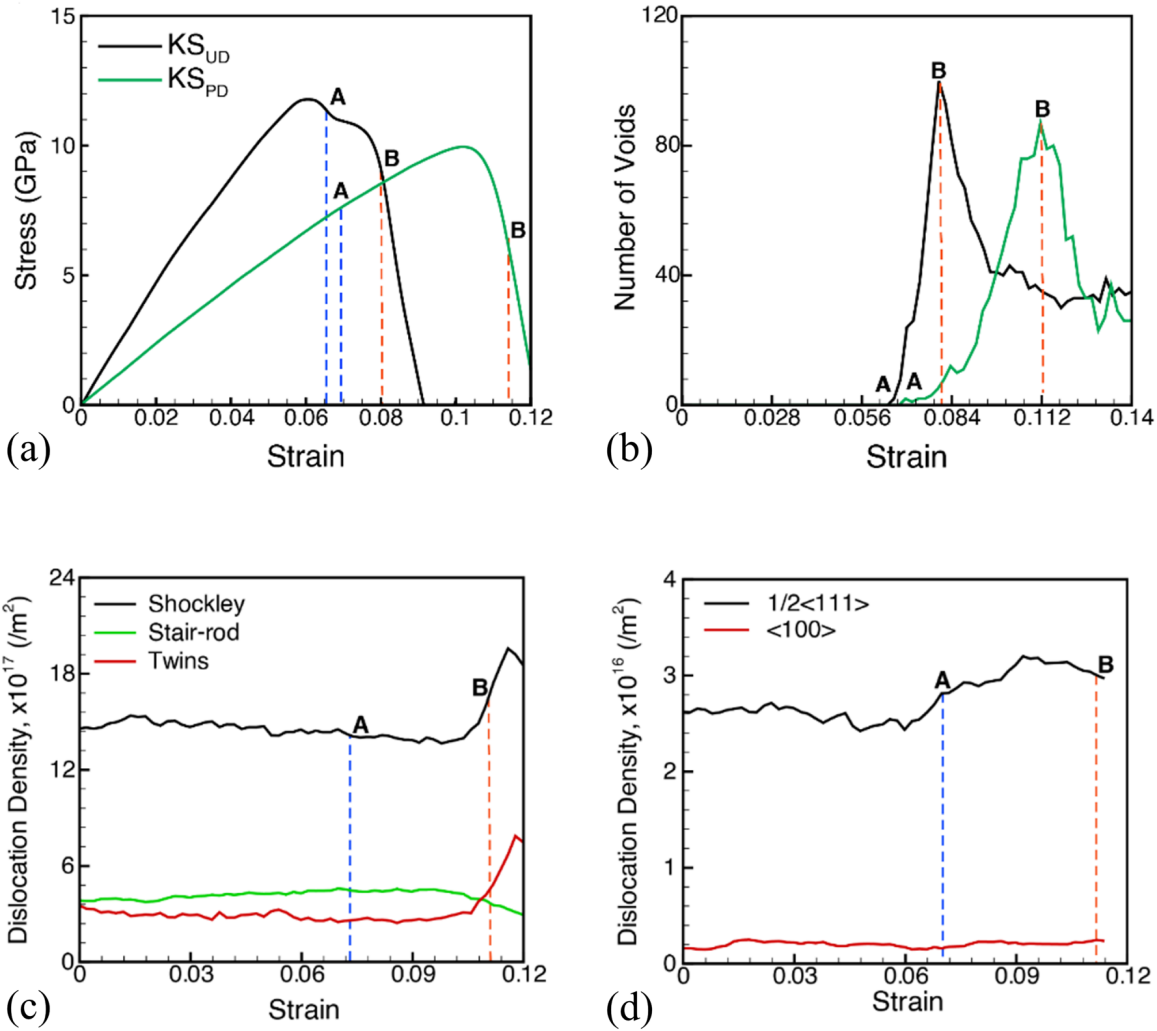
Plots for (**a**) stress–strain behavior and (**b**) evolution of the number of voids during the uniaxial expansion of the un-deformed and pre-deformed KS interfaces loaded in the direction perpendicular to the interface. The blue and orange dashed vertical lines represent the void nucleation and peak number of voids points, respectively. Dislocation density evolution in the pre-deformed interface system during uniaxial expansion as observed in (**c**) the Cu layer and (**d**) the Ta layer.

The as-created KS Cu/Ta system is deformed under uniaxial strain compression with a strain rate of 10^9^ s^−1^ normal to the interface up to a strain of 0.02 (20%) to mimic the effect of shock compression. This strain results in a pressure of 60 GPa for the Cu/Ta system. The compressed system is then unloaded back to zero pressure to mimic the system after shock release. While no twins are observed in the Ta slab after unloading to zero pressure, dislocations are observed in the Cu layers. This pre-deformed system is then deformed under conditions of uniaxial tensile strain at a strain rate of 10^9^ s^−1^. A comparison of the stress–strain curve during the uniaxial expansion of the pre-deformed system with that of the un-deformed Cu/Ta KS system is plotted in Fig. 3a. The pre-deformed interface system has a reduced spall strength as compared to the un-deformed system. The evolution of voids during the expansion of the pre-deformed system is plotted in Fig. 3b in comparison with the undeformed system. The stresses at which void nucleation is observed, i.e., the minimum void nucleation strain, is similar in both cases. However, void nucleation in the undeformed case is observed beyond a peak in the stresses in the stress–strain curve. In contrast, void nucleation for the pre-deformed case is accompanied by continued strain hardening after void nucleation (point A). However, the strain at the peak number of voids is delayed significantly for the pre-deformed system compared to the undeformed system. Thus, a longer void nucleation stage is observed for the pre-deformed system compared to the un-deformed system and includes an increase in stresses to a peak value followed by a decrease before the onset of the growth and coalescence stage. Even after void nucleation in the pre-deformed system, the continued strain hardening behavior is likely due to modifications in the dislocation density evolution behavior for the pre-deformed cases as plotted in Fig. 3c, d for the Cu and Ta layers, respectively. The uniaxial expansion of the pre-deformed systems from an initial compression strain of 20% with a high initial dislocation density does not lead to an additional increase in the density of dislocations. The softening of the undeformed Cu/Ta KS system after nucleation of voids is attributed to the sharp rise in dislocation density during the nucleation stage of voids (point A to point B). The continued strain hardening behavior of the pre-deformed Cu/Ta system after nucleation of voids is therefore likely due to the inability to nucleate addition dislocations in the Cu layer and a very small increase in the Ta layer during the nucleation stage of voids. A sharp increase in dislocation density is observed in the Cu layer just before the end of the nucleation stage and coincides with a drop in the stresses in Fig. 3a**.** It should be noted that although the pre-deformation resulted in the distribution of dislocations (an order of magnitude lower) in the Ta layer, no voids were nucleated in the Ta layer.

**Figure 4:**
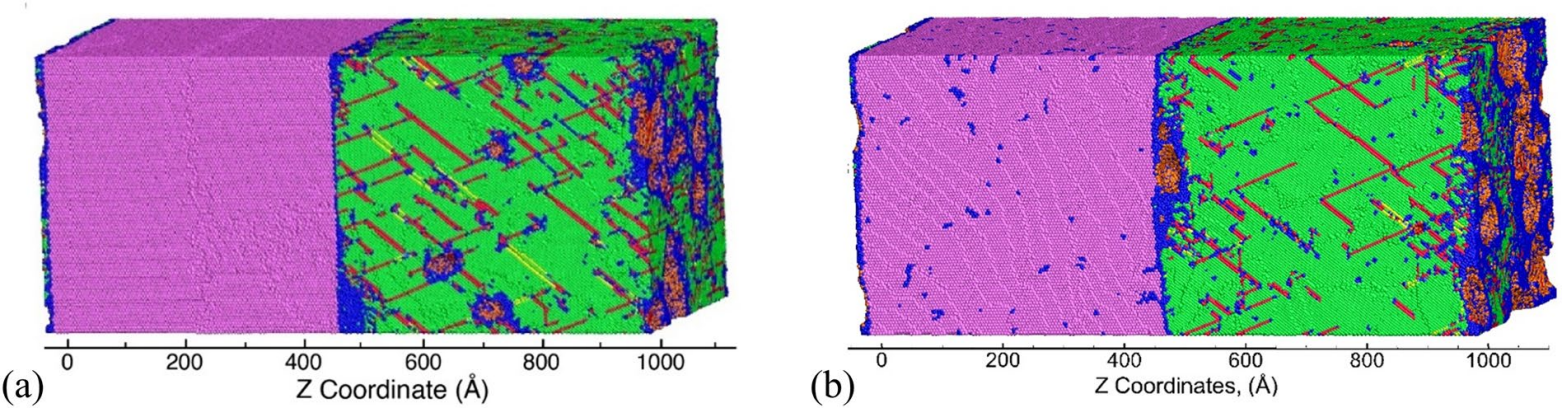
Snapshot showing the void distribution at point B. Here, purple, green, blue, red, and orange color atoms represent Ta-BCC, Cu-FCC, disordered, stacking fault, and Cu/Ta surface/void atoms, respectively.

These modifications in the dislocation density evolution behavior result in modifications in the damage nucleation behavior for the two systems. The microstructure at the time of the peak number of voids is shown in Fig. 4a, b for the undeformed and pre-deformed systems, respectively. Here, the purple, green, blue, red, and orange color atoms represent Ta-BCC, Cu-FCC, disordered, stacking fault, and Cu/Ta surface/void atoms, respectively. While the snapshot of the undeformed system shows voids nucleating in the Cu layer at the interface and at stacking fault intersections inside the Cu layer ^47,48^, the snapshot of the pre-deformed system shows voids nucleating mostly at the Cu/Ta interface in the Cu layers.

#### Plasticity contributions during void nucleation and growth/coalescence

During this simulation, a thorough analysis of activated slip systems for loading perpendicular to the KS interface is performed for the Cu and Ta slabs. Since plastic deformation is not observed in the Ta slab, the slip system analysis is limited to the evolution of dislocations and faults in the Cu slab. The atomic fractions (%) of stacking fault atoms belonging to different slip systems in the fcc Cu slab for the undeformed system are tabulated in Table 1 at Point A (void nucleation) and point B (peak number of voids) in Fig. 2a. The three primary slip systems in the KS-Cu/Ta system i.e. $\left(\overline{1 }11\right)$, $(1\overline{1 }1)$, and $(11\overline{1 })$ have equal Schmid factors (SF) with an equal probability of activation. However, out of three primary slip systems, the ($11\overline{1 })[112]$ slip system is activated first because it lies along the direction of the misfit dislocation line (dashed black line, as shown in Fig. 1b). Hence, the activation of slip systems in layered materials is not solely a function of the SF but can also be affected by the alignment of interfacial misfit dislocations. If the direction of any of the slip system is aligned with the misfit dislocations, there are higher chances that that slip system contributes more to the dislocation plasticity. The slip systems that contribute to the distribution of stacking fault atoms along the interface are shown in Fig. 2b. The plasticity contributions at later stages (peak number of voids), however, are nearly equal in all three primary slip systems, as tabulated in Table 1. In comparison, the atomic fractions (%) of stacking fault atoms belonging to different slip systems in the fcc Cu slab at Point A and at point B for the pre-deformed system are also tabulated in Table 1**.** Pre-deformation, therefore, has a minimal effect on the plasticity contributions from the activated slip systems as compared to the undeformed system. The modifications are, therefore, primarily in the stresses and strains for the nucleation stage of voids.

**Table 1:** Stacking fault atom fractions (%) of the four fcc slip systems in Cu at the void nucleation strain (Point A), ${SF}_{N}^{void}$, and at peak void number strain (Point B), ${SF}_{M}^{void}$, for loading in the direction perpendicular to the KS interface in the undeformed and pre-deformed system.

Slip plane	Undeformed Cu/Ta		Pre-deformed Cu/Ta	
	${SF}_{N}^{void}$ (%)	${SF}_{M}^{void}$ (%)	${SF}_{N}^{void}$ (%)	${SF}_{M}^{void}$ (%)
$(111)$	0^S, 0^	0^S, 0^	7^S, 0^	1^S, 0^
$(\overline{1 }11)$	18^P, 0.31^	36^P, 0.31^	22^P, 0.31^	34^P, 0.31^
$(1\overline{1 }1)$	21^P, 0.31^	35^P, 0.31^	17^P, 0.31^	28^P, 0.31^
$(11\overline{1 })$	62^P, 0.31^	28^P, 0.31^	54^P, 0.31^	37^P, 0.31^

Here, superscripts P and S denote the slip systems (P—primary, S—secondary) along with their SF values for corresponding loading orientations.

#### Effect of loading parallel to the KS interface on void nucleation behavior

**Figure 5:**
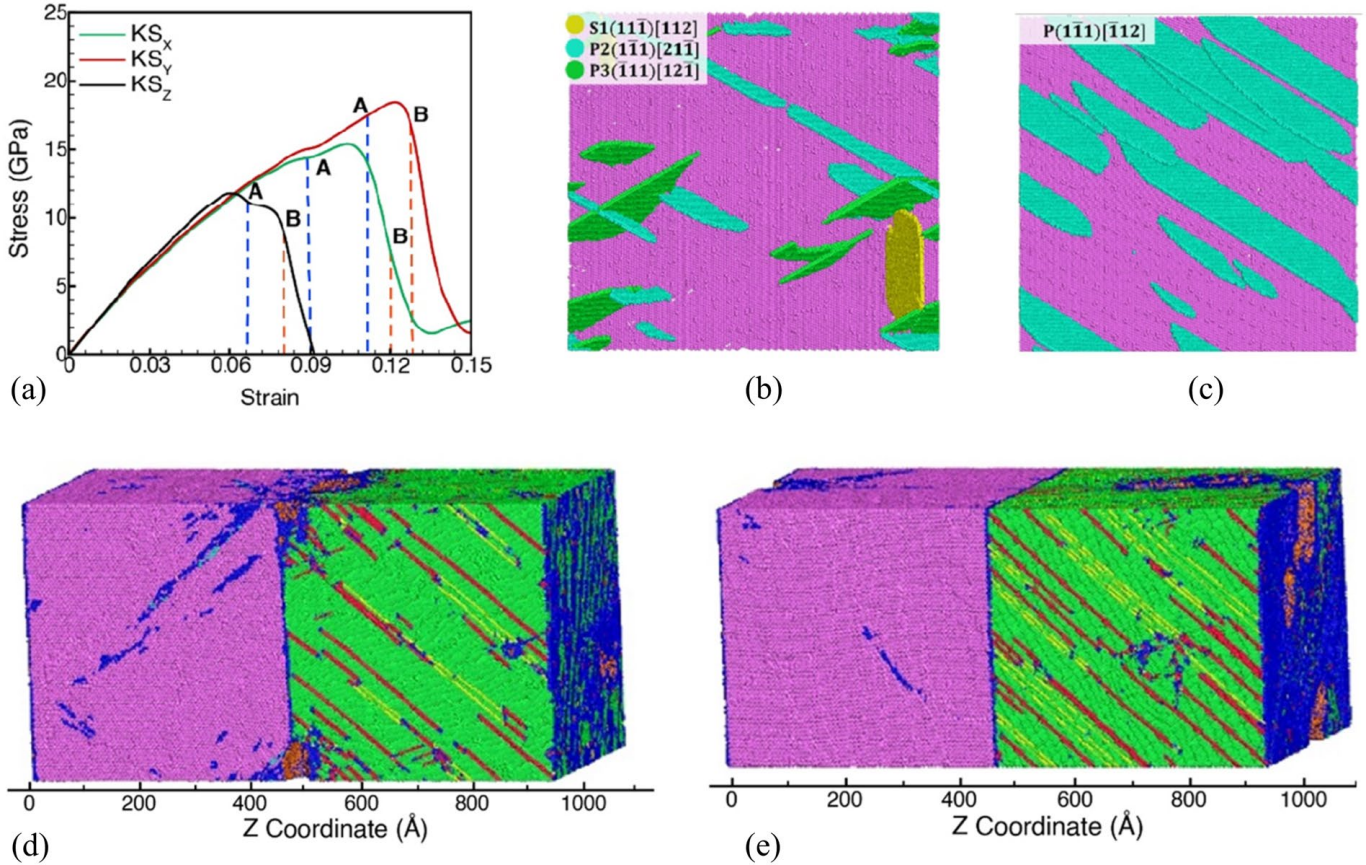
(**a**) Stress–strain plots for X [1$\overline{1 }\! \overline{2 }$], Y $[1\overline{1 }0]$and Z [111] loading orientations for the KS interface. Here, blue and orange dashed vertical lines at points A and B represent the void nucleation and peak number of voids points, respectively. Snapshots of the KS interface showing the contributions from the slip systems when loaded in (**b**) the X direction and (**c**) the Y direction. The system snapshots show void distributions at point B when loaded in (**b**) the X direction and (**c**) the Y direction.

MD simulations are also carried out for loading in the direction parallel to the KS interface to investigate the effect on the plasticity contributions and the nucleation stage of voids. The stress–strain plots for loading in the $[\overline{1 }\overline{1 }2]$_Cu_ (X) direction and the $[1\overline{1 }0]$_Cu_ (Y) direction parallel to the KS interface are plotted in Fig. 5a. The spall strength in the parallel directions (X, and Y) is considerably higher than that in the normal (Z) direction for the previously discussed KS interface. The higher strength is attributed to the co-deformation of the Ta layer with the Cu layer, which affects the void nucleation stresses in the Cu layers. The slip systems that contribute to plasticity for loading parallel to the interface by the distribution of stacking fault atoms along the interface are shown in Fig. 5b, c for loading along the X and Y directions, respectively. The corresponding atomic fractions (%) of stacking fault atoms belonging to different slip systems in the Cu slab at Point A and point B for the two parallel loading orientations are tabulated in Table 2 compared to that in the normal direction. For loading along the Y direction, although two slip systems have the same SF value of 0.47, the slip is dominated by systems aligned along the misfit dislocation line in the corresponding direction of the concerned slip system. In comparison to direction Y where the plasticity contribution is dominated by the slip systems aligned along misfit dislocation lines, the plasticity contributors for loading in the X direction are determined by a combination of the high value of SF (0.39) and the alignment of the slip system along misfit dislocations. The two slip systems in the X direction have equal values and both have a significant contribution to plasticity. The system snapshots showing the distribution of voids at point B are shown in Fig. 5d, e for loading along the X and Y directions, respectively. Voids nucleate mostly at the Cu/Ta interface in the Cu layers in both loading directions. A comparison of the evolution of the number of voids, void volume fraction, and density of dislocations in the Cu and Ta layers when loaded in the parallel direction with that loaded in the perpendicular direction is plotted in Fig. S2 of Supplemental Information.

**Table 2:** Stacking fault atom fractions (%) of the four fcc slip systems in Cu at the void nucleation strain (Point A), ${SF}_{N}^{void}$, and at peak void number strain (Point B), ${SF}_{M}^{void}$, for different loading directions in the KS interface in the undeformed and pre-deformed system.

Slip plane	${SF}_{N}^{void}$ (%)			${SF}_{M}^{void}$ (%)		
	KS_X	KS_Y	KS_Z	KS_X	KS_Y	KS_Z
$(111)$	0^S, 0^	0^S, 0^	0^S, 0^	3^S, 0^	0.6^S, 0^	0^S, 0^
$(\overline{1 }11)$	63^P, 0.39^	0.4^P, 0.47^	18^P, 0.31^	54^P, 0.39^	1.6^P, 0.47^	36^P, 0.31^
$(1\overline{1 }1)$	29^P, 0.39^	99.6^P, 0.47^	21^P, 0.31^	39^P, 0.39^	96.4^P, 0.47^	35^P, 0.31^
$(11\overline{1 })$	8^S, 0.31^	0^S, 0^	62^P, 0.31^	4^S, 0.31^	1.4^S, 0^	28^P, 0.31^

Here, superscripts P and S denote the slip systems (P—primary, S—secondary) along with their SF values for corresponding loading orientations.

The evolution of the number of voids and void fractions for all the systems of KS interface (perpendicular/parallel loading cases of KS_**X**_, KS_**Y**_, KS_**Z**_ and preformed KS_**Z-PD**_) is analyzed to compare the calculated values for the void nucleation stress (${\sigma }_{V}^{N}$), void nucleation strain (${\varepsilon }_{V}^{N.}$), total dislocation density at void nucleation (${\rho }_{V}^{N}$), total dislocation density at peak number of voids (${\rho }_{V}^{M}$), as well as the stress and strain values at peak number of voids (${\sigma }_{V}^{M}$, ${\varepsilon }_{V}^{M.}$) during uniaxial strain expansion of the Cu/Ta system with the KS interface in the direction perpendicular (Z) and parallel to the KS interface (X, Y) in Table 3 and includes the pre-deformed systems. It can be observed from Table 3 that the stresses and strains for void nucleation decrease as the total density of dislocations in the system at the time of void nucleation increases. The highest void nucleation stresses are observed for loading in the direction parallel to the KS interface along the Y direction and has the lowest number for the density of dislocations. Similarly, the lowest stresses for void nucleation are observed for the pre-deformed KS interface in the perpendicular direction and is correlated with the highest density of dislocations in the system.

**Table 3:** The calculated values for total dislocation density at void nucleation (${\rho }_{V}^{N}$), dislocation density at the peak number of voids (${\rho }_{V}^{M}$), loading stress at void nucleation (${\sigma }_{V}^{N}$), loading strain at void nucleation (${\varepsilon }_{V}^{N}$), loading stress at the peak number of voids (${\sigma }_{V}^{M}$), and loading strain at the peak number of voids (${\varepsilon }_{V}^{M}$), during uniaxial strain expansion of the KS interface system in the direction perpendicular (Z) and parallel to the KS interface (X, Y).

Loading	${\rho }_{V}^{N}$ × 10^17^	${\rho }_{V}^{M}$ × 10^17^	${\sigma }_{V}^{N}$(GPa)	${\varepsilon }_{V}^{N}$ (%)	${\sigma }_{V}^{M}$(GPa)	${\varepsilon }_{V}^{M}$(%)
KS_X_	13.7	15.8	14.5	9.2	13.6	11.2
KS_Y_	5.2	13.5	17.4	11.6	16.5	12.8
KS_Z_	18.1	30.8	11.4	6.6	9.1	8.0
KS_Z-PD_	21.9	25.8	7.7	6.9	7.8	11.2

### Effect of orientation relationships on void nucleation behavior

Six Cu/Ta interfaces with different ORs (as tabulated in Table S1 of the Supplemental Information) are investigated here to understand the role of loading orientation and slip systems. The stress–strain curves for the six systems are plotted in the Fig. S3 of the Supplementary Information. The values for the stresses, strains, and dislocation densities at point A (void nucleation) and point B (peak number of voids) for the various interface systems are tabulated in Table 4. The spall strength values are the lowest for the interface systems with the highest interface energy and highest for the systems with the lowest energies of the interface.

**Table 4:** The calculated values for total dislocation density at void nucleation (${\rho }_{V}^{N}$), dislocation density at the peak number of voids (${\rho }_{V}^{M}$), loading stress at void nucleation (${\sigma }_{V}^{N}$), loading strain at void nucleation (${\varepsilon }_{V}^{N}$), loading stress at the peak number of voids (${\sigma }_{V}^{M}$), and loading strain at the peak number of voids (${\varepsilon }_{V}^{M}$), during uniaxial strain expansion of the various interface systems in the direction perpendicular (Z) to the interface.

OR	${\rho }_{V}^{N}$ (× 10^17^)	${\rho }_{V}^{M}$ (× 10^17^)	${\sigma }_{V}^{N}$ (GPa)	${\varepsilon }_{V}^{N}$ (%)	${\sigma }_{V}^{M}$ (GPa)	${\varepsilon }_{V}^{M}$ (%)
KS	18.1	30.8	11.4	6.6	9.1	8.0
NW	18.3	26.5	12.3	7.0	9.1	7.8
KS112	19.3	25.6	10.6	6.8	8.9	8.2
OT3	7.5	12.5	10.1	6.7	9.7	8.0
OT1	8.8	23.2	10.1	7.2	8.3	9.2
OT2	8.9	23.6	11.2	7.0	9.6	8.4

Among the six systems, it is observed that the Cu/Ta systems with the same direction of loading for the Cu slab show a similar kind of stress–strain behavior. For example, the loading perpendicular to the KS and NW interface results in the Cu slab deforming along the [111] direction and showing similar spall strength values. Similarly, loading perpendicular to the KS112 and OT3 interfaces renders deforming the Cu slab along the [112] orientation, and loading perpendicular to the OT1 and OT2 interfaces renders deforming the Cu slab along the $\left[1\overline{1 }0\right]$ loading orientation. Hence, three representative interface systems (KS, KS112, and OT1) are discussed in detail to investigate the effect of loading orientations of the Cu slab. The dashed-black lines mark the distribution of interface misfit dislocations, and the nucleation of stacking faults at the KS112 and OT1 interfaces is shown in the Fig. S4 of the Supplementary Information. It can be observed that the secondary slip system ($11\overline{1 })[112]$ following the misfit dislocation lines is activated first for the KS112 interfaces, whereas the primary slips system $\left(\overline{1 }11\right)[1\overline{1 }2]$ and $\left(1\overline{1 }1\right)[\overline{1 }12]$ are activated first at the OT1 interface according to SF analysis rather than following the misfit dislocation criterion. This may be attributed to the very high SF of 0.47 for these slip systems.

**Figure 6:**
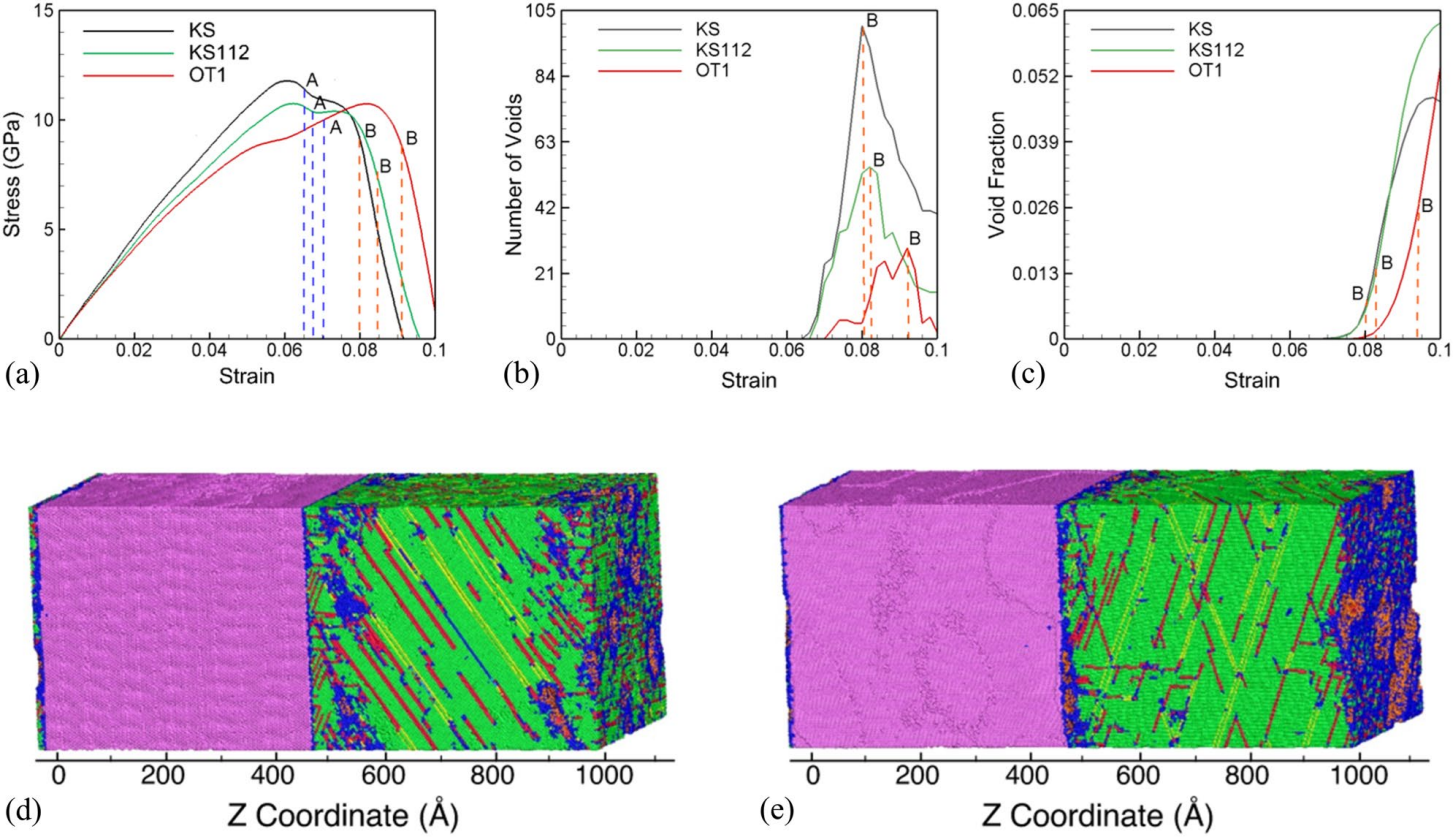
(**a**) Stress–strain response of KS, KS112 and OT1 interface systems in [111]**,** [112] and [110] loading directions, respectively. The evolution of (**b**) number of voids and (**c**) void fraction in KS, KS112 and OT1 interface structures. Here, blue and orange dashed vertical lines at points A and B represent the void nucleation and peak number of voids points, respectively. System microstructure at the time of maximum voids for the (**d**) in KS112 interface system, and (**e**) in the OT1 interface system. Here, purple, green, blue, red and orange atoms represent Ta-BCC, Cu-FCC, disordered atoms, stacking faults, Ta surface atoms, and voids in Cu, respectively.

Figure 6a compares the stress–strain curves for the three systems during uniaxial strain expansion. The three different orientations for the Cu slab in these systems show differences in the stress–strain curves and, more importantly, after nucleation of voids (dashed blue lines). The KS interface system, as discussed before, shows a continuous decrease in stress during the nucleation stage of voids. The KS112 interface system, in comparison, shows a very slight increase (or a plateau) after void nucleation. In contrast, the OT1 interface system shows strain hardening and a delayed nucleation stage for damage evolution. The void nucleation stress is highest for the KS interface system among the three interface systems. In addition, the evolution of the void fraction and the number of voids for the three interface systems are shown in Fig. 6b, c, respectively. The KS interface system has the highest number of voids, and the OT1 has the lowest number of voids at point B (orange dashed lines). The void fraction values at the time of peak number of voids suggest that the KS interface has the smallest average size of the voids, KS112 has intermediate, and OT1 has the largest void size. Thus, the interfaces with higher energy values nucleate larger voids than those with lower energy values. As a result, growth and coalescence seem to be more prevalent in higher energy interfaces than lower energy interfaces where nucleation of multiple voids is more dominant. The snapshots of system microstructure for KS112 and OT1 interfaces at point B are shown in Fig. 6d, e, respectively. The snapshots show that most of the voids are located at the interfaces in the Cu layer. However, some voids are also observed in the interior of the Cu slabs at stacking fault intersections.

**Figure 7:**
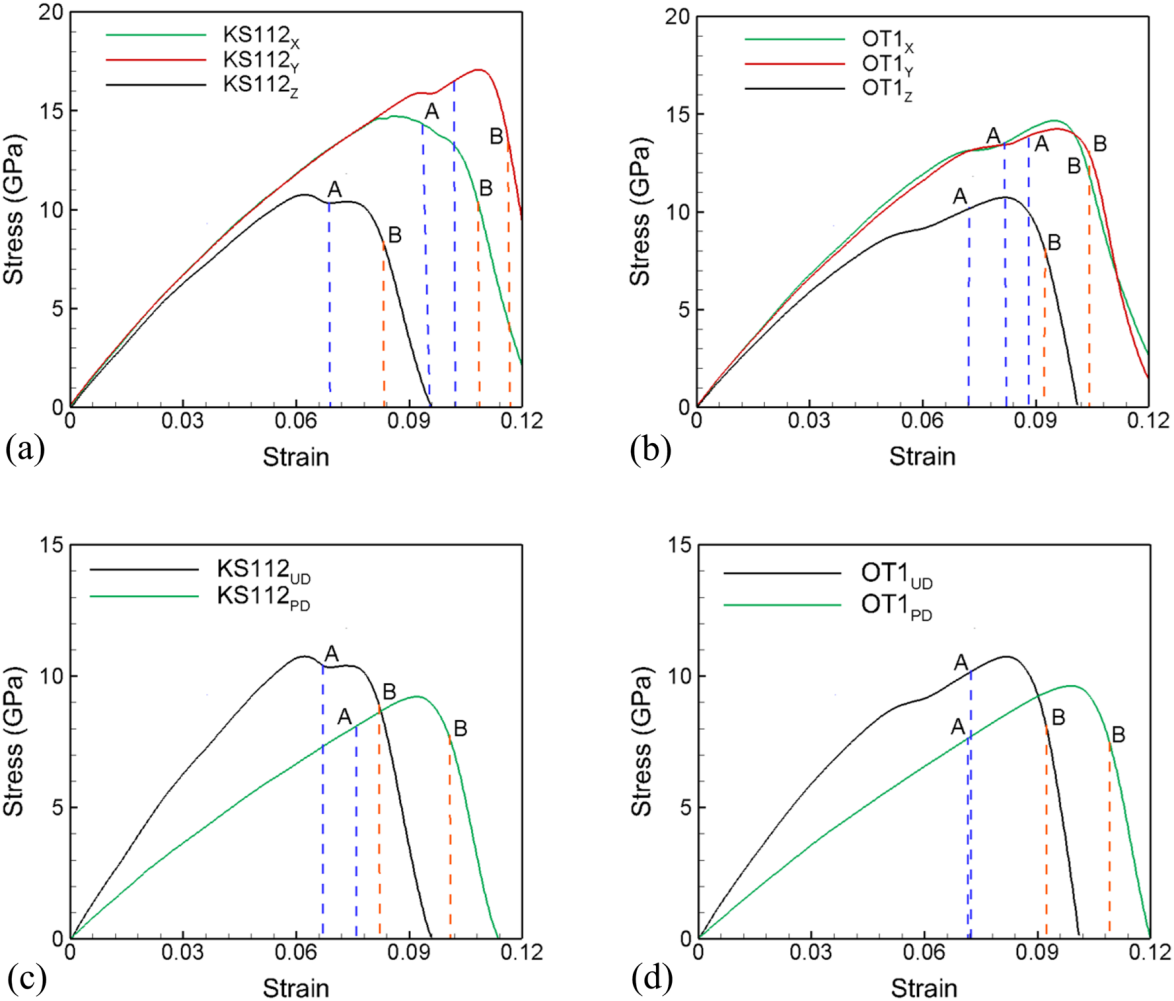
Comparison of stress–strain plots for *X*[1̅1̅1], *Y*[11̅0], and *Z*[112] loading orientations in (**a**) KS112 interface system, and (**b**) OT1 interface system. Comparison of stress–strain plot for un-deformed and pre-deformed (**c**) KS112 and (**d**) OT1 interfaces loaded in the direction perpendicular to the interface. Blue and orange dashed vertical lines represent the void nucleation and peak number of voids points, respectively. Here, blue and orange dashed vertical lines at points A and B represent the void nucleation and peak number of voids points, respectively.

The three interface systems are also investigated under uniaxial strain tensile loading conditions in the direction parallel (X, Y) to the interface. The corresponding stress–strain plots for loading in the X and Y directions are plotted in Fig. 7a, b for KS112 and OT1 interface systems, respectively, and compared with that in the Z direction. As observed for the KS interface, the void nucleation stresses are considerably higher for loading parallel to the interface for KS112 and OT1 interface systems. The corresponding evolution of void fraction and number of voids for loading in directions parallel to the interface is plotted for the KS112 system in Fig. S5 and for the OT1 systems in the Fig. S6 of Supplementary Information. While deformation is observed in the Ta slabs for loading in parallel directions, the Ta layers do not nucleate voids. SF analysis is therefore used to understand the plasticity contributors at the time of nucleation of voids and at the peak number of voids. A detailed comparison of the atomic fraction (%) of stacking fault atoms belonging to four FCC slip systems in Cu at void nucleation and peak number void points is tabulated for the three loading orientations for the KS112 in Table S2 of and for the OT1 interface systems in the Table S3 of the Supplementary Information. For the case of the KS112 interface system loaded along the X direction, even though the secondary slip system has zero SF, it contributes most towards activated slip because it lies along with the interface misfit dislocation lines. However, at the time of the peak number of voids, the contribution from the secondary slip system diminishes, and primary slip systems contribute toward most of the slip. A simple geometrical analysis is observed for Y orientation, and secondary slip systems with zero SF are absent from slip activation because of very high SF 0.47 for primary slip planes. For the case of the OT1 interface system loaded along both X and Y orientations, no-slip system lies along the misfit dislocation lines. Hence, a geometrical consideration is followed in activation of various slip planes in Y orientation of KS112 interface where only the primary slip systems with higher SF factors are activated.

The values for stresses, strains, and dislocation densities at points A and B for the KS112 and the OT1 system are tabulated in Tables S4 and S5 in the Supplementary Information. It can be inferred from these tables that dislocation and void nucleation starts at higher values of stress and strains for parallel loading directions in KS112 and OT1 interfaces. The stresses and strains for void nucleation in the KS112 system vary inversely with the total density of dislocations in the system at the time of void nucleation. The highest void nucleation stresses are observed for loading in the direction parallel to the KS interface along the Y direction, and has the lowest number for the density of dislocations. Similarly, the lowest stresses for void nucleation are observed for loading along the perpendicular direction and has the highest density of dislocations in the system. However, no such correlation is observed for the OT1 interface system.

The six interface systems are also pre-deformed to a strain of 20% under a uniaxial compressive strain at a strain rate of 10^9^ s^−1^ in the normal direction to mimic shock compression. The compressed systems are then unloaded back to zero pressure resulting in dislocations distributed in the microstructure. The pre-deformed systems are then loaded under uniaxial strain conditions at a constant strain rate of 10^9^ s^−1^. While deformation is observed in the Ta layers, the twin fraction and dislocation density is very small as compared to that in the Cu layers. As observed with the pre-deformed KS interface, all the other pre-deformed interface systems render reduced failure strengths compared to un-deformed cases and also show increased strain hardening and duration of the void nucleation stage. Example stress–strain curves for pre-deformed KS112 and OT1 interfaces are shown in Fig. 7c, d, respectively. The pre-deformation results in a reduced spall strength as compared to the un-deformed systems for all the interface systems and results in increased strains for void nucleation.

In addition, the effect of pre-deformation on the slip system activation and atomic fractions belonging to different slip systems are calculated and tabulated in Table S6 of the Supplementary Information. It can be observed from the tables that the pre-deformation does not have any appreciable effect on the activation/suppression of slip systems in the pre-deformed structures. However, the pre-deformed structures have some contribution of slip from secondary slip systems such as $(111)$ at point A, which was absent in un-deformed structures. However, the primary slip systems produce most of the stacking faults at the peak number voids point.

The comparison of the evolution of void fraction and number of voids of pre-deformed KS112 and OT1 systems with undeformed systems is plotted in Fig. S7 of the Supplementary Information. In pre-deformed cases, KS and KS112 have a lower number of peak voids than OT1. The evolution of the number of voids and void fractions for all systems is analyzed to compare the calculated values for dislocation densities, stresses, and strains at points A and B for the pre-deformed systems and tabulated in the Table S7 of the Supplementary Information. The spall strength has the highest value for the KS112 orientation and the lowest value for the OT1 orientation for the pre-deformed systems. In contrast, the spall strength value is highest for the KS orientation and lowest for the OT1 orientation in the undeformed interface systems (Table 3). However, this highest value of the pre-deformed KS112 system is also due to the with the lowest dislocation density at point A.

**Figure 8:**
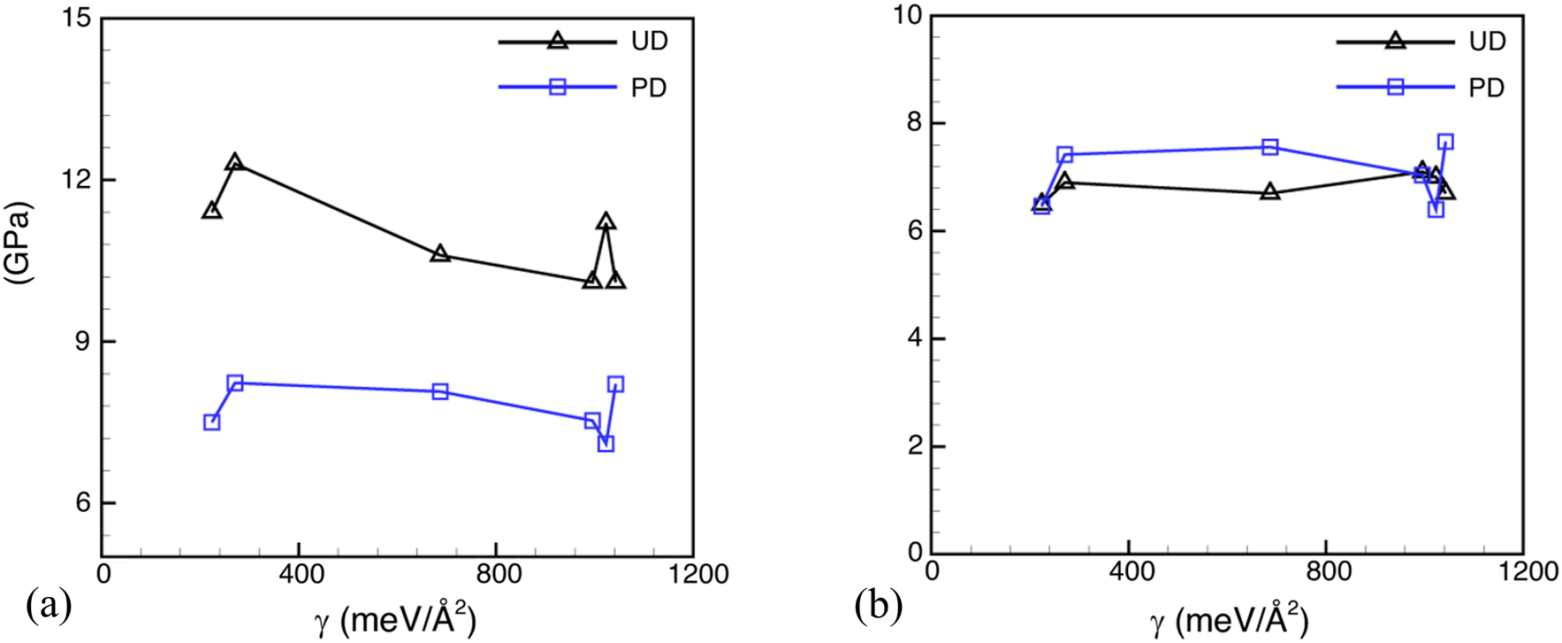
Variation of (**a**) void nucleation stress and (**b**) strain with interface energy in un-deformed and pre-deformed cases for loading in perpendicular direction.

Further, to investigate the dependence of void nucleation stress, ${\sigma }_{V}^{N}$, and strain, ${\varepsilon }_{V}^{N}$, on the interface for un-deformed and pre-deformed systems, these quantities have been plotted against interface energy ($\gamma )$ in Fig. 8. The results suggest that as the interface energy increases void nucleation stresses is observed to decrease in undeformed systems. This relationship, however, is not observed for pre-deformed systems. It can be therefore considered that interface energy does not play a significant role in determining the void nucleation stresses for the interface microstructures under dynamic loading conditions.

## Conclusions

Large-scale MD simulations investigate the effect of various Cu/Ta interface microstructures on the damage nucleation behavior by investigating the six interface systems with different loading orientations. The simulations use loading conditions of uniaxial expansion for as-created as well as pre-deformed microstructures. The MD simulations identify a nucleation stage and a growth/coalescence stage of voids for all the microstructures considered. The activation of slip systems in these systems is observed to be not solely a function of the SF but can also be affected by the alignment of interfacial misfit dislocations.

For all the systems loaded perpendicular to the interface, plasticity is only observed in the Cu phase. Pre-deformation of the system does not significantly affect the plasticity contributions from the activated slip systems as compared to the undeformed system The failure of the as-created KS interface system is accompanied by a softening behavior due to a sharp rise in dislocation density in the Cu layer during the nucleation stage of voids. In contrast, the failure of the pre-deformed KS interface system is accompanied by strain hardening behavior due to the inability to nucleate addition dislocations in the Cu layer and a very small increase in the Ta layer during the nucleation stage of voids. For the as-created KS interface system, the nucleation stage is accompanied by a softening behavior in the stress–strain curve. Voids nucleate in the Cu slabs at the Cu/Ta interfaces as well as in the interior of the Cu layer. In contrast, the as-created KS112 interface system shows a very slight increase (or a plateau) in the stress–strain curve after void nucleation and the OT1 interface system shows strain hardening during the nucleation stage. Hence, the damage nucleation stage is determined by the loading orientation of the Cu slab. The spall strength values are the lowest for the interface systems (KS112 and OT1) with the highest interface energy and highest for the systems with the lowest energy of the interface (KS). The stresses to nucleate voids in Cu are considerably lower for the interface microstructures that undergo pre-deformation compared to as-created systems. For systems loaded parallel to the interface, the co-deformation of Cu and Ta, along with different loading orientations of Ta, helps in improving the overall strength of the Cu/Ta system. The void nucleation stresses are considerably higher for loading parallel to the interface as compared to loading perpendicular to the interface. While deformation is observed in the Ta slabs for loading in the parallel directions, the Ta slabs do not nucleate any voids. Irrespective of the loading orientation, the spall strength values for as-created systems are the lowest for the interface systems (KS112 and OT1) with the highest interface energy and highest for the systems with the lowest energy of the interface (KS). This correlation, however, is not observed for pre-deformed systems.

## Materials and method

### Molecular dynamics (MD)

MD simulations use the open source code LAMMPS^56^ using the “angular dependent potential” (ADP)^50^ to describe the interatomic interactions. This study investigates the role of interface structure, considering the six Cu/Ta interfaces, on the dislocation/void nucleation mechanisms and the corresponding stresses for dislocation/void nucleation.

### Cu/Ta microstructures

Model microstructures of Cu/Ta choose the three most common experimentally observed interfaces with lower energy, i.e., Kurdjumov–Sachs (KS)^47^, Nishiyama-Wassermann (NW)^52^, and KS112^53^, along with three less commonly observed higher interface energy interfaces. The details of all these six interfaces, along with respective interface energy values, are provided in Table of Note 1 in the Supplemental Information. Each interface represents a specific orientation relation (OR). For example, KS has an OR, Cu {111} <110>||Ta {110}  <111>, which means the Cu (111) plane is parallel to Ta (110) plane and direction $[11\overline{0 }]$ in Cu is parallel to the direction [111] in Ta. For the KS in Fig. 1a, Cu [111] and Ta $[11\overline{0 }]$ are aligned along Z-axis, while Cu $[11\overline{0 }]$ and Ta [111] are along the Y-axis. All other interfaces also share a similar kind of model structure. For convenience, the rest of the three interfaces have been named other-1 (OT1), other-2 (OT2), and other-3 (OT3), as indicated in *Table* of *Note 1* in the *Supplemental Information*. The initial relaxed structures for the six interfaces are shown in *Figure* of *Note 1* in the *Supplemental Information*. Flat interfaces such as KS show initial dislocations with an in-plane Burger vector, while faceted interfaces like KS112 have dislocations extending out of the interface plane with an out-of-plane Burger vector component. Each system has dimensions of ~ 39 nm × ~ 39 nm × ~ 96 nm along X, Y, and Z directions, respectively, as shown in Fig. 1a, and is comprised of ~ 10 million atoms. Prior to the deformation, every structure is equilibrated at 300 K and zero pressure using the NPT ensemble using the Nose–Hoover thermostat and barostat as implemented in LAMMPS. Periodic boundary conditions are employed in all three principal directions i.e., X, Y, and Z.

### Cu/Ta interface energy calculations

The energy of each interface is calculated using Eq. (1)

1 $$\gamma ={\frac{1}{A}(E}_{Cu/Ta}-{N}_{Cu}{E}_{Coh/Cu}-{N}_{Ta}{E}_{Coh/Ta})$$

where, ${E}_{(\mathrm{Cu}/\mathrm{Ta})}$ is the total energy of the Cu/Ta system, and ${E}_{Coh/Cu}$, and ${E}_{Coh/Ta}$ are the cohesive energies of Cu and Ta in the FCC and BCC structures, respectively; ${N}_{Cu}$ and ${N}_{Ta}$, represent the number of Cu and Ta atoms in the Cu/Ta system, respectively, being considered, and A is the interface area. The computed values of interface energy for each interface are tabulated in the Table of Note 1 in the Supplemental Information. The Misfit dislocation arrays in the Figure of Note 1 in the Supplemental Information are the intrinsic characteristics of these interfaces ^44,47,57^ and usually govern the deformation behavior of these interfaces under various loading conditions.

### Deformation conditions and characterization of defects and voids

To mimic the dynamic shock loading conditions, the deformation of the system is carried out under uniaxial strain conditions at a constant strain rate of 10^9^ s^−1^ and with a time step of 2 fs. In experimental shock loading conditions, the section of the material experiencing spall failure a sample first undergoes uniaxial strain compression and then under uniaxial strain tension (referred to as uniaxial expansion) that results from the interaction of reflected waves to initiate spall failure^26,58^. Hence MD simulations are carried out first for loading conditions of uniaxial strain tension (referred to as uniaxial expansion). The conditions of uniaxial strain loading refer to strains being applied only in one direction and the strains in the lateral direction are zero. This results in a state of triaxial stress i.e. the stress in all the lateral directions are also non-zero. For example, loading under uniaxial strain expansion or compression loading along the Z direction results in stress states corresponding to ${\varepsilon }_{\text{z}}\ne 0, {\varepsilon }_{\text{x}}={\varepsilon }_{\text{y}}=0$ and ${\sigma }_{\text{z}}\ne 0, {\sigma }_{\text{y}}\ne 0, {\sigma }_{\text{x}}\ne 0$. In addition, MD simulations are also carried out for loading conditions of uniaxial strain compression (referred to as uniaxial compression) to a strain of 20%, equilibrated for 50 ps (NVE), and then unloaded to zero pressure to create pre-deformed microstructures for all the systems are also considered. The pre-deformed systems are then subjected to uniaxial tensile strain. The stress–strain plots are created using the Virial stress formulation^59^, and the generated defects (stacking faults, twin faults, dislocations, etc.) are analyzed using a combination of “centrosymmetric parameter” (CSP)^60^, “common neighbor analysis” (CNA)^61,62^, “dislocation extraction algorithm” ^63,64^ (DXA) using OVITO^65^ and “the crystal analysis tool” (CAT)^66^. The characterized FCC dislocations include perfect dislocations (1/2  <110>), Shockley partial 1/6 <112>, Frank partial (1/3 <111>), Hirth locks (1/3  <001>), stair-rods (1/6  <110>) and twinning partial dislocations 1/6  <112> in Cu. For BCC Ta, dislocations with Burgers vectors 1/2  <111>,  <100>, and  <110>  are characterized. More details about these characterization methodologies can be found elsewhere^47,67–69^. The void analysis is based on superimposing the MD snapshots at any time in 3D cells and identifying clusters of empty connected cells as voids^26^.

## Supplementary Information

Supplementary Information.

## Data Availability

The datasets used and/or analysed during the current study available from the corresponding author on reasonable request.
