# Supplementary material for: Interface microstructure effects on dynamic failure behavior of layered Cu/Ta microstructures
Source: Sci Rep. 2023 Jul 13;13:11365. doi: 10.1038/s41598-023-37831-5 (PMC10344864; doi:10.1038/s41598-023-37831-5)
Supplement: Supplementary file 1 — Supplementary Information. [file 41598_2023_37831_MOESM1_ESM.docx]

Supporting Information

**Interface Microstructure Effects on Dynamic Failure Behavior of Layered Cu/Ta Microstructures**

**Authors:** Rajesh Kumar^1^, Jie Chen^1^, Avanish Mishra^1^, and Avinash M. Dongare^1^

**Affiliation:**

*^1^Department of Materials Science and Engineering, Institute of Materials Science, University of Connecticut, 97 North Eagleville Road, Storrs, CT 06269, USA*

* **Corresponding Author:**

Avinash M. Dongare

- **Address:** Department of Materials Science and Engineering & Institute of Materials Science, 97 North Eagleville Road, Unit 3136 Storrs, CT 06269-3136, USA
- **Email:** [dongare@uconn.edu](mailto:dongare@uconn.edu)
- **Telephone:** +1 860-486-2592

TABLES AND TABLE CAPTIONS

**Table S1:** Different types of Cu/Ta interfaces with their specific orientation relationships and interface energy values at 0 K. Here, OT1, OT2, and OT3 stand for other-1, other-2 and other-3 interfaces, respectively.

| Interface | Orientation Relationship (OR) | Interface Energy γ (mJ/m^2^) |
| --- | --- | --- |
| KS | Cu$\left( 111 \right)\left[ 1\bar{1}0 \right]$ \|\| Ta$\left( 1\bar{1}0 \right) \left[ 111 \right]$ | 223.98 |
| NW | Cu$\left( 111 \right)\left[ 1\bar{1}0 \right]$ \|\| Ta$\left( 110 \right) \left[ 001 \right]$ | 270.53 |
| KS112 | Cu$\left( 112 \right)\left[ \bar{1}\bar{1}1 \right]$ \|\| Ta$\left( 112 \right)\left[ 1\bar{1}0 \right]$ | 686.96 |
| OT1 | Cu$\left( 1\bar{1}0 \right)\left[ 111 \right]$ \|\| Ta$\left( 001 \right) \left[ 1\bar{1}0 \right]$ | 995.58 |
| OT2 | Cu$\left( 1\bar{1}0 \right)\left[ 111 \right]$\|\| Ta$\left( 111 \right) \left[ 1\bar{1}0 \right]$ | 1023.10 |
| OT3 | Cu$\left( \bar{1}\bar{1}2 \right)\left[ 111 \right]$ \|\| Ta$\left( 001 \right) \left[ 110 \right]$ | 1042.66 |

**Table S2:** Atomic fraction (%) of the stacking fault atoms belonging to four fcc slip systems in Cu at void nucleation (${SF}_{N}^{void}$), i.e., at Point A, and peak number void points (${SF}_{M}^{void}$), i.e., at Point B, for different loading orientations in the KS112 interface system. Here, P and S stand for primary and secondary slip systems, while the numbers in superscripts are the value of the Schmid factor for that slip system for corresponding loading orientations.

| Slip Plane | $\boldsymbol{SF}_{\boldsymbol{N}}^{\boldsymbol{void}}$, (%) | | | $\boldsymbol{SF}_{\boldsymbol{M}}^{\boldsymbol{void}}$, (%) | | |
| --- | --- | --- | --- | --- | --- | --- |
|  | KS112_X_ | KS112_Y_ | KS112_Z_ | KS112_X_ | KS112_Y_ | KS112_Z_ |
| $\boldsymbol{(111)}$ | 24^P, 0.31^ | 0^S, 0^ | 1^S, 0.31^ | 23^P, 0.31^ | 4^S, 0^ | 7^S, 0.31^ |
| $\boldsymbol{(}\bar{\boldsymbol{1}}\boldsymbol{11)}$ | 19^P, 0^*^.31^* | 47^P, 0.47^ | 37^P, 0.39^ | 27^P, 0.31^ | 45^P, 0.47^ | 40^P, 0.39^ |
| $\boldsymbol{(1}\bar{\boldsymbol{1}}\boldsymbol{1)}$ | 26^P, 0.31^ | 52^P, 0.47^ | 38^P, 0.39^ | 38^P, 0.31^ | 47^P, 0.47^ | 46^P, 0.39^ |
| $\boldsymbol{(11}\bar{\boldsymbol{1}}\boldsymbol{)}$ | 31^S, 0^ | 1^S, 0^ | 62^P, 0.31^ | 12^S, 0^ | 4^S, 0^ | 7^S, 0^ |

**Table S3**: Atomic fraction (%) of stacking fault atoms belonging to four fcc slip systems in Cu at void nucleation (${SF}_{N}^{void}$) i.e., at Point A, and peak number void points (${SF}_{M}^{void}$) i.e. at Point B, for different loading orientations in the OT1 interface system. Here, P and S stand for primary and secondary slip systems, while the numbers in superscripts are the value of the Schmid factor for that slip system for corresponding loading orientations.

| Slip Plane | $\boldsymbol{SF}_{\boldsymbol{N}}^{\boldsymbol{void}}$, (%) | | | $\boldsymbol{SF}_{\boldsymbol{M}}^{\boldsymbol{void}}$, (%) | | |
| --- | --- | --- | --- | --- | --- | --- |
|  | OT1_X_ | OT1_Y_ | OT1_Z_ | OT1_X_ | OT1_Y_ | OT1_Z_ |
| $\boldsymbol{(111)}$ | 0^S, 0^ | 0^S, 0^ | 0^S, 0^ | 5^S, 0^ | 1.5^S, 0^ | 12^S, 0^ |
| $\boldsymbol{(}\bar{\boldsymbol{1}}\boldsymbol{11)}$ | 29^P, 0.31^ | 61^P, 0.39^ | 76^P, 0.47^ | 37^P, 0.31^ | 64.5^P, 0.39^ | 64^P, 0.47^ |
| $\boldsymbol{(1}\bar{\boldsymbol{1}}\boldsymbol{1)}$ | 26^P, 0.31^ | 39^P, 0.39^ | 24^P, 0.47^ | 29^P, 0.31^ | 33^P, 0.39^ | 15^P, 0.47^ |
| $\boldsymbol{(11}\bar{\boldsymbol{1}}\boldsymbol{)}$ | 45^P, 0.31^ | 0^S, 0.31^ | 0^S, 0^ | 29^S, 0.31^ | 1.0^S, 0.31^ | 8^S, 0^ |

**Table S4:** The calculated values for total dislocation density at void nucleation ($\rho_{V}^{N}$), dislocation density at the peak number of voids ($\rho_{V}^{M}$), loading stress at void nucleation ($\sigma_{V}^{N}$), loading strain at void nucleation ($\varepsilon_{V}^{N}$), loading stress at the peak number of voids ($\sigma_{V}^{M}$), and loading strain at the peak number of voids ($\varepsilon_{V}^{M}$), during uniaxial strain expansion of the Cu/Ta system with the KS112 interface in the direction perpendicular (Z) and parallel to the interface (X, Y).

| OR | $\rho_{V}^{N}$  (x10^17^) | $\rho_{V}^{M}$  (x10^17^) | $\sigma_{V}^{N}$  (GPa) | $\varepsilon_{V}^{N}$  (%) | $\sigma_{V}^{M}$  (GPa) | $\varepsilon_{V}^{M}$  (%) |
| --- | --- | --- | --- | --- | --- | --- |
| KS112_X_ | 17.3 | 34.1 | 14.3 | 9.4 | 10.5 | 10.8 |
| KS112_Y_ | 12.6 | 35.0 | 16.5 | 10.2 | 13.8 | 11.6 |
| KS112_Z_ | 19.3 | 25.6 | 10.6 | 6.8 | 8.9 | 08.2 |

**Table S5:** The calculated values for total dislocation density at void nucleation ($\rho_{V}^{N}$), dislocation density at peak number of voids ($\rho_{V}^{M}$), loading stress at void nucleation ($\sigma_{V}^{N}$), loading strain at void nucleation ($\varepsilon_{V}^{N}$), loading stress at peak number of voids ($\sigma_{V}^{M}$), and loading strain at peak number of voids ($\varepsilon_{V}^{M}$), during uniaxial strain expansion of the Cu/Ta system with the OT1 interface in the direction perpendicular (Z) and parallel to the interface (X, Y).

| OR | $\rho_{V}^{N}$  (x10^17^) | $\rho_{V}^{M}$  (x10^17^) | $\sigma_{V}^{N}$  (GPa) | $\varepsilon_{V}^{N}$  (%) | $\sigma_{V}^{M}$  (GPa) | $\varepsilon_{V}^{M}$  (%) |
| --- | --- | --- | --- | --- | --- | --- |
| OT1_X_ | 14.7 | 31.8 | 13.6 | 8.2 | 12.0 | 10.4 |
| OT1_Y_ | 10.9 | 14.5 | 13.9 | 8.8 | 13.0 | 10.4 |
| OT1_Z_ | 8.7 | 23.2 | 10.1 | 7.2 | 1-8.3 | 9.2 |

**Table S6:** Atomic fraction (%) of *the* stacking fault atoms belonging to four fcc slip systems in Cu at void nucleation *(*$\mathrm{SF}_{N}^{\mathrm{void}}$*) i.e., at Point A,* and peak number void points *(*$\mathrm{SF}_{M}^{\mathrm{void}}$*)* i.e.*,* at Point B, for different loading orientations that are initially un-deformed and pre-deformed. Here, P and S stand for primary and secondary slip systems, while the numbers in superscripts are the value of the Schmid factor for that slip system for corresponding loading orientations.

| Slip Plane | $\boldsymbol{SF}_{\boldsymbol{N}}^{\boldsymbol{void}}$, (%) | | | $\boldsymbol{SF}_{\boldsymbol{M}}^{\boldsymbol{void}}$, (%) | | |
| --- | --- | --- | --- | --- | --- | --- |
|  | KS/KS_PD_ | KS112/KS112_PD_ | OT1/OT1_PD_ | KS/KS_PD_ | KS112/KS112_PD_ | OT1/OT1_PD_ |
| $\boldsymbol{(111)}$ | 0/7^S, 0^ | 1/7^S, 0.31^ | 0/10^S, 0^ | 0/1^S, 0^ | 7/3^S, 0.31^ | 12/16^S, 0^ |
| $\boldsymbol{(}\bar{\boldsymbol{1}}\boldsymbol{11)}$ | 18/22^P, 0.31^ | 37/62^P, 0.39^ | 76/51^P, 0.47^ | 36/34^P, 0.31^ | 40/65^P, 0.39^ | 64/41^P, 0.47^ |
| $\boldsymbol{(1}\bar{\boldsymbol{1}}\boldsymbol{1)}$ | 21/17^P, 0.31^ | 38/27^P, 0.39^ | 24/33^P, 0.47^ | 35/28^P, 0.31^ | 46/24^P, 0.39^ | 15/31^P, 0.47^ |
| $\boldsymbol{(11}\bar{\boldsymbol{1}}\boldsymbol{)}$ | 62/54^P, 0.31^ | 24/4^S, 0^ | 0/6^S, 0^ | 28/37^P, 0.31^ | 7/8^S, 0^ | 8/12^S, 0^ |

**Table S7:** The calculated values for total dislocation density at void nucleation ($\rho_{V}^{N}$), dislocation density at peak number of voids ($\rho_{V}^{M}$), loading stress at void nucleation ($\sigma_{V}^{N}$), loading strain at void nucleation ($\varepsilon_{V}^{N}$), loading stress at peak number of voids ($\sigma_{V}^{M}$), and loading strain at peak number of voids ($\varepsilon_{V}^{M}$), during uniaxial strain expansion of the pre-deformed Cu/Ta systems with the KS, KS112, and OT1 interfaces in the direction perpendicular (Z) to the interface.

| System | $\rho_{V}^{N}$  x10^17^ | $\rho_{V}^{M}$  x10^17^ | $\sigma_{V}^{N}$  (GPa) | $\varepsilon_{V}^{N}$  (%) | $\sigma_{V}^{M}$  *(GPa)* | $\varepsilon_{V}^{M}$  (%) |
| --- | --- | --- | --- | --- | --- | --- |
| KS_PD_ | 20.5 | 25.8 | 7.7 | 0.069 | 7.8 | 0.112 |
| KS112_PD_ | 14.9 | 16.9 | 8.1 | 0.075 | 8.2 | 0.099 |
| OT1_PD_ | 19.9 | 26.4 | 7.5 | 0.07 | 7.8 | 0.108 |

FIGURES AND FIGURE CAPTIONS


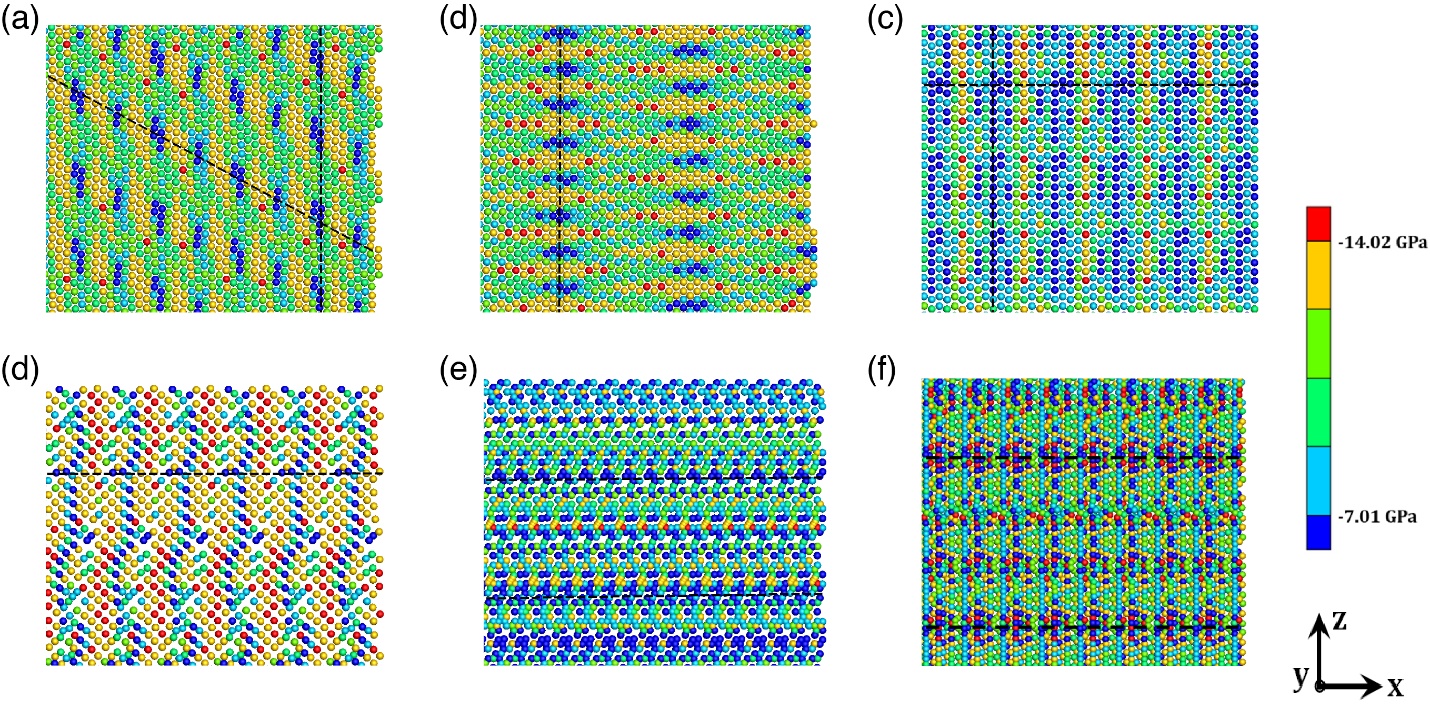


**Figure S1:** Top view of various interfaces showing misfit dislocations, here, (a), (b), (c), (d), (e)

and (f) represent KS, NW, KS112, OT1, OT2 and OT3, interfaces, respectively. The atoms have

been colored based on the Z stress component. Here the dashed line shows the distribution of misfit dislocations at the interface.

**
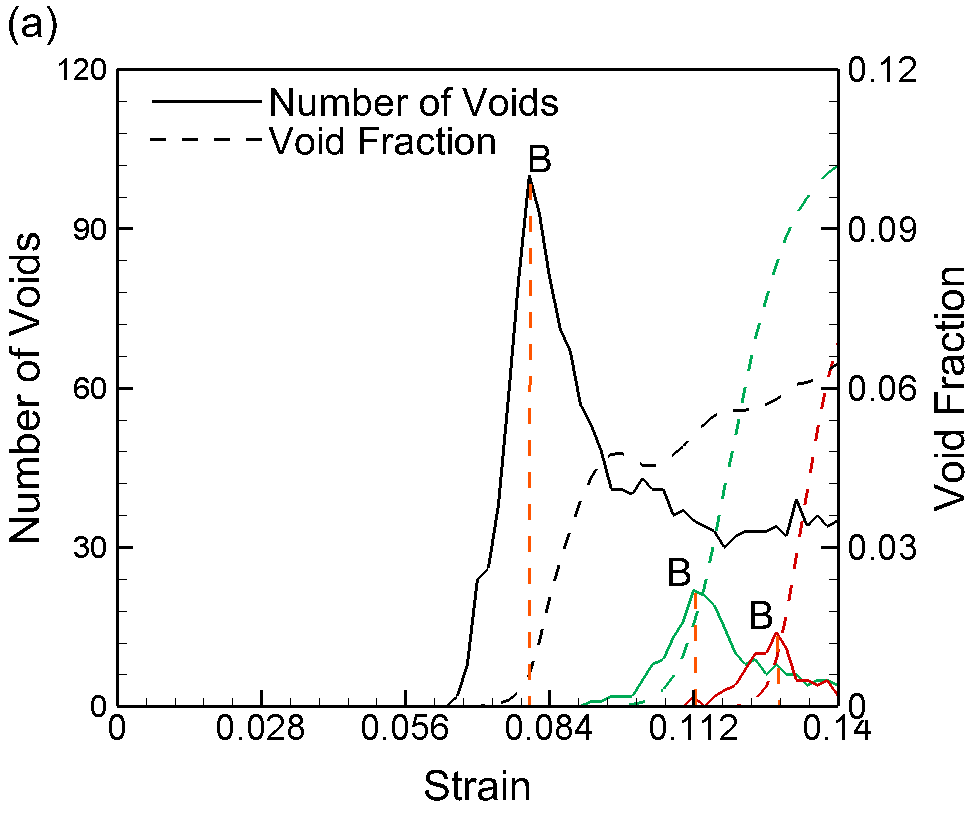

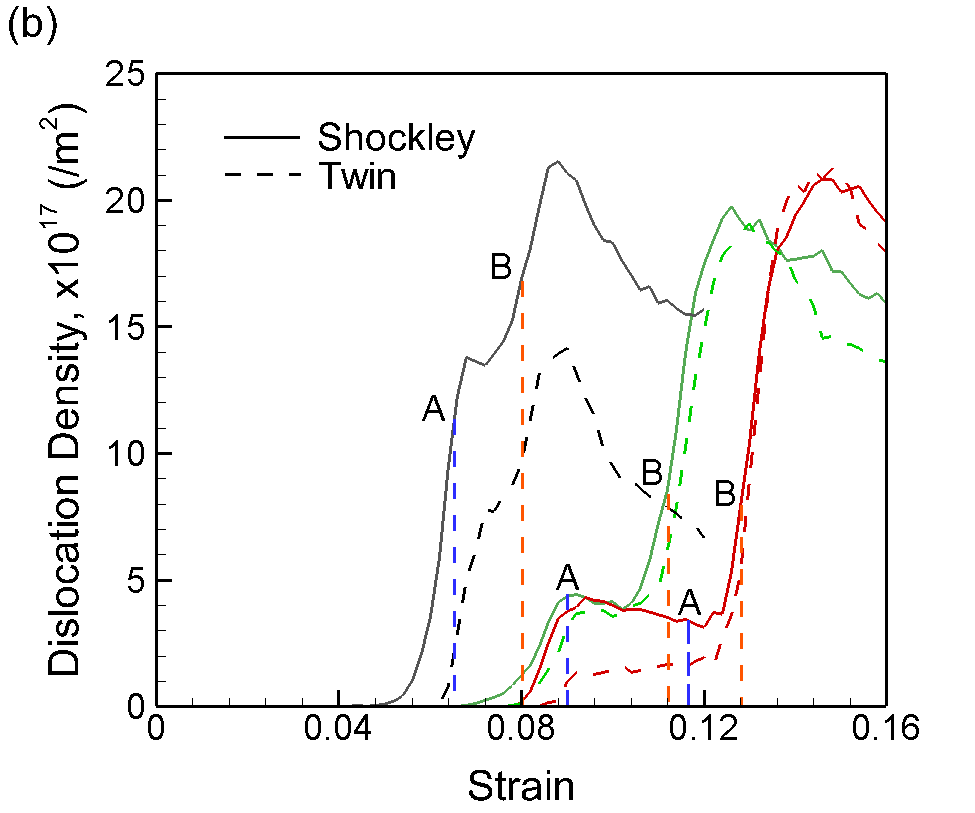
**

**Figure S2:** Comparison of the evolution of, (a) the number of voids and void fraction and, (b) the evolution of dislocation density, as functions of applied strain for parallel and normal to the interface loading directions for KS interface. Here green, red and black curves represent KS_X_, KS_Y_ and KS_Z_ directions, respectively.


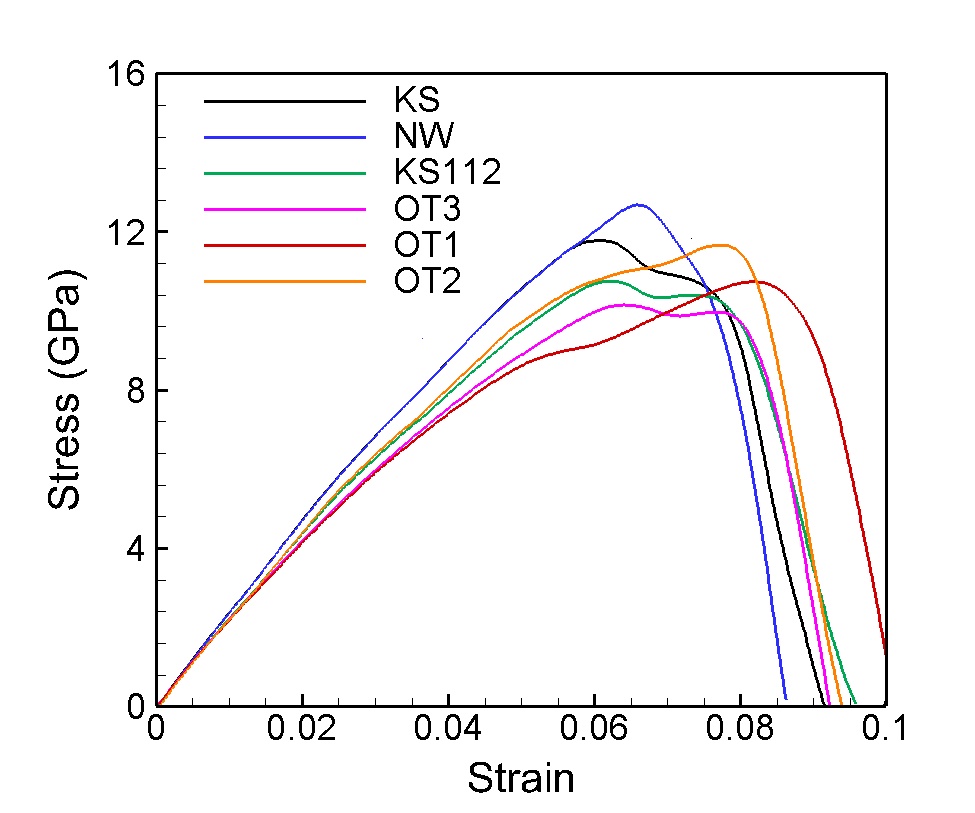


**Figure S3**: Stress-strain plot for various interface systems when loaded in the direction perpendicular (Z) to the interface.

|  | 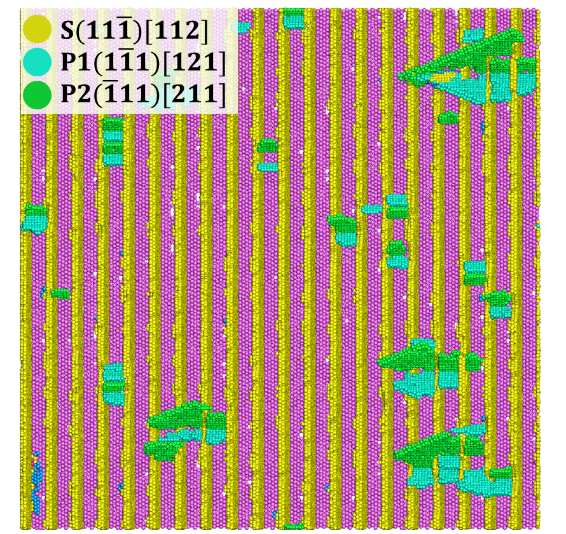  (b) |
| --- | --- |
|   (a)  (c) | 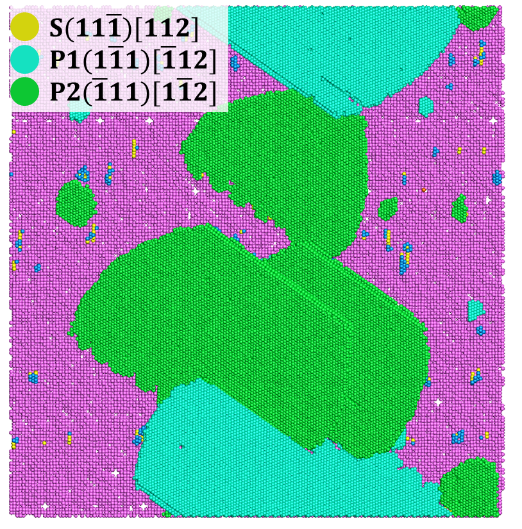  (d) |

**Figure S4:** Nucleation of stacking faults in for (a) and (b) KS112, and (c) and (d) OT1 interfaces, respectively. Black dashed line represents the line of misfit dislocations.


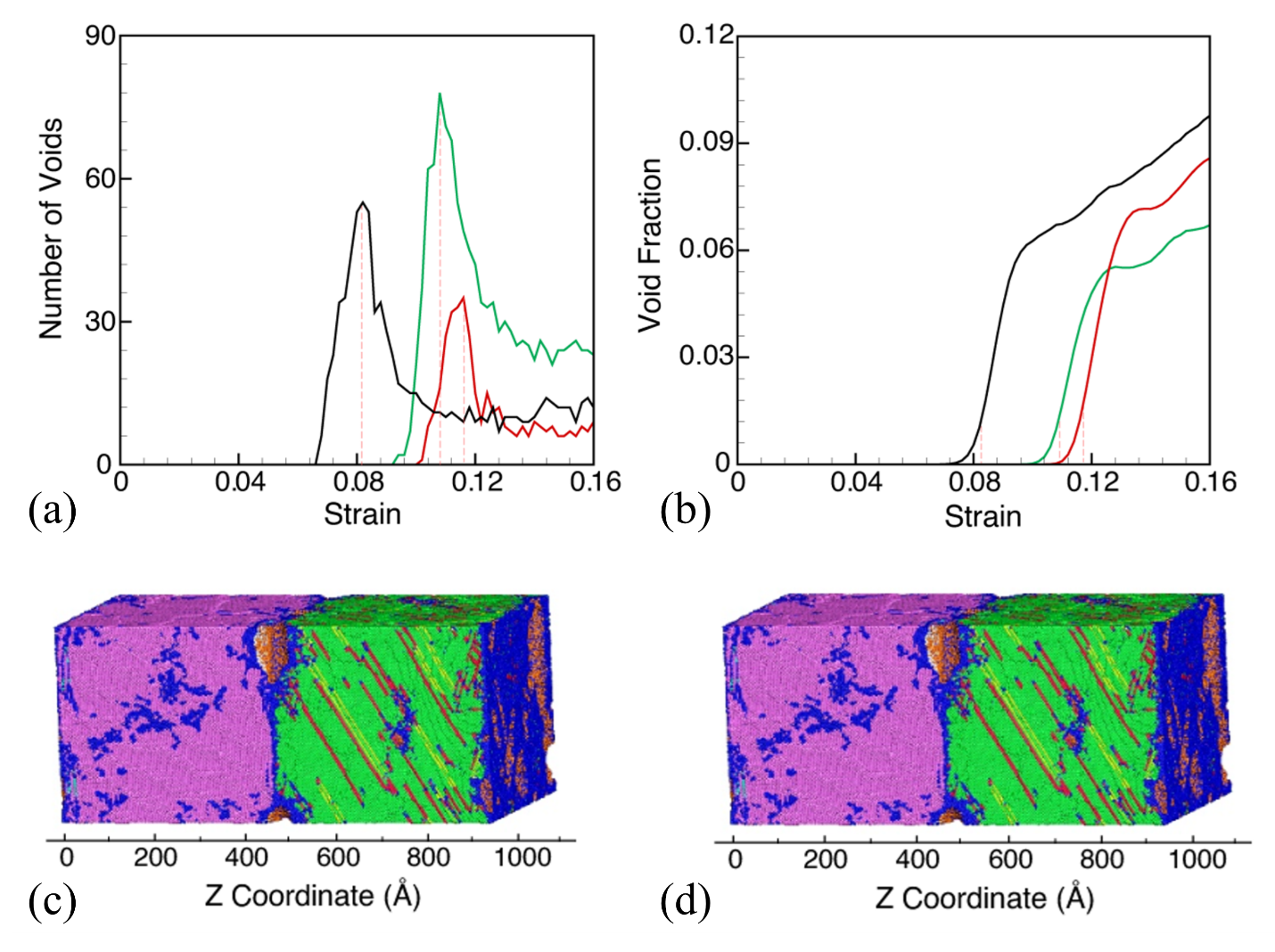


**Figure S5:** Evolution of (a) number of voids and (b) void fraction for 𝑋 $\bar{[1}\bar{1}1]$, Y [1$\bar{1}0]$, and 𝑍 [112] loading orientations in KS112 interface. Blue and orange dashed vertical lines represent the void nucleation and peak number of voids points, respectively. System microstructure at the time of maximum voids for loading in (c) in X direction, and (d) in the Y direction for the KS112 interface system. Here, purple, green, blue, red and orange atoms represent Ta-BCC, Cu-FCC, disordered atoms, stacking faults, Ta surface atoms, and voids in Cu, respectively.

**
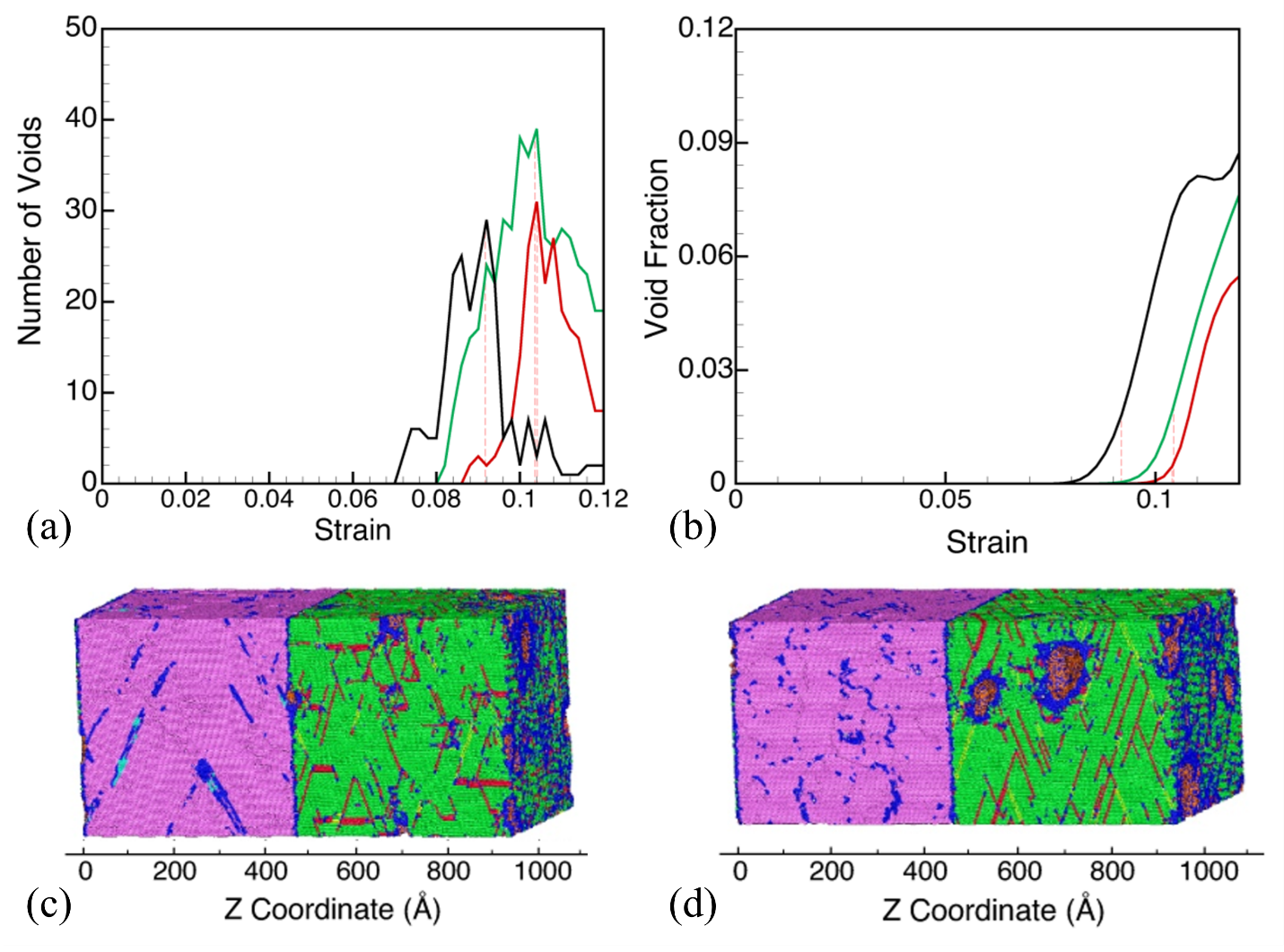
**

**Figure S6:** Evolution of (a) number of voids and (b) void fraction for 𝑋 $\bar{[1}\bar{1}2]$, Y $[1\bar{1}0]$, and 𝑍 [111] loading orientations in OT1 interface. Orange dashed vertical lines represent the void nucleation and peak number of voids points, respectively. System microstructure at the time of maximum voids for loading in (c) in X direction, and (d) in the Y direction for the OT1 interface system. Here, purple, green, blue, red and orange atoms represent Ta-BCC, Cu-FCC, disordered atoms, stacking faults, Ta surface atoms, and voids in Cu, respectively.


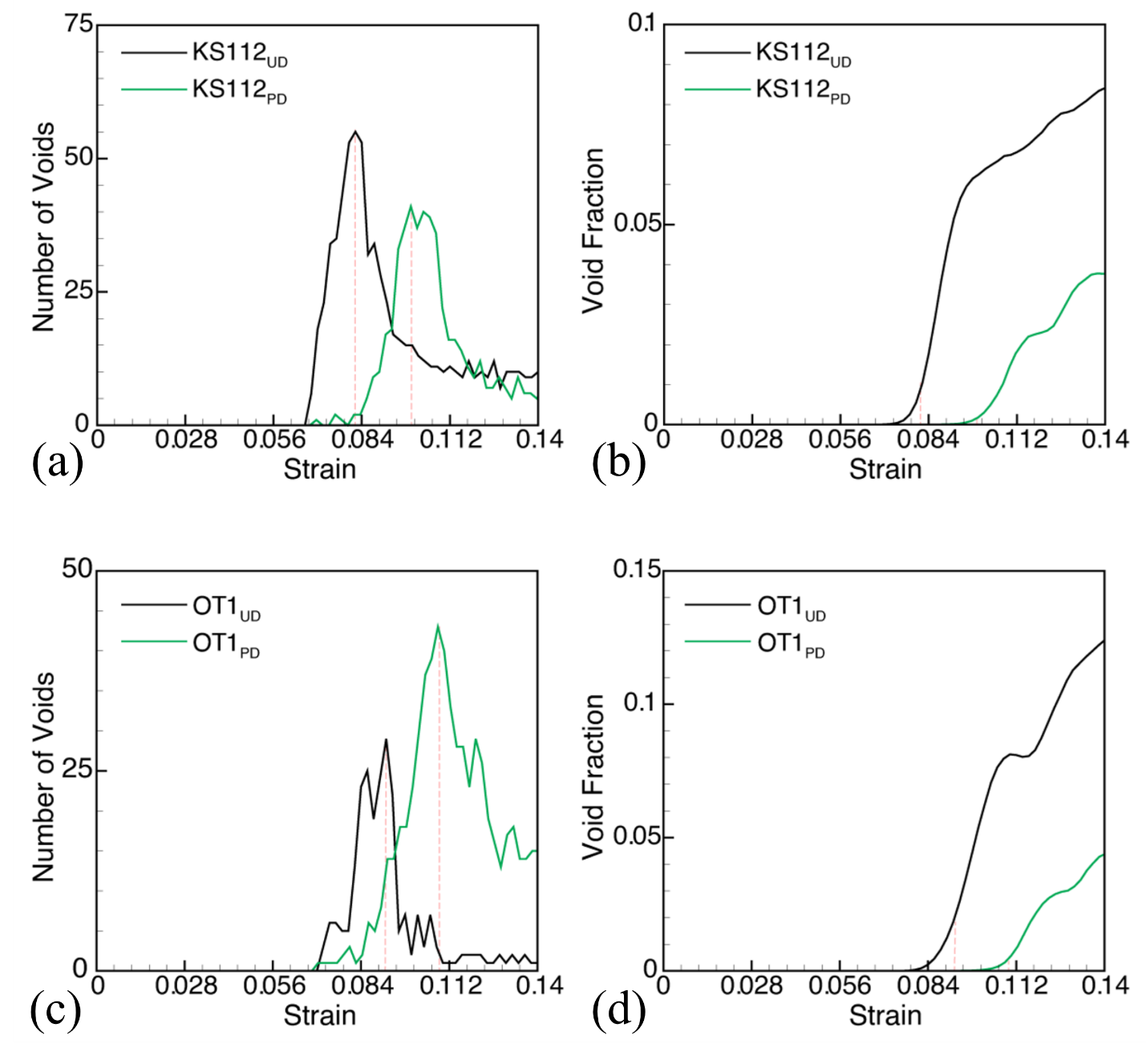


**Figure S7:** The evolution of the number of voids (a) and (c), and void fraction (b) and (d) for un-deformed and pre-deformed structures of KS112 and OT1, respectively. Blue and orange dashed vertical lines represent the void nucleation and peak number of voids points, respectively.
